# The Histopathological Correlate of Peri-Vascular Adipose Tissue Attenuation on Computed Tomography in Surgical Ascending Aorta Aneurysms: Is This a Measure of Tissue Inflammation?

**DOI:** 10.3390/diagnostics11101799

**Published:** 2021-09-29

**Authors:** Nicola Gaibazzi, Domenico Tuttolomondo, Francesco Nicolini, Alessandro Tafuni, Daniele Sartorio, Chiara Martini, Francesco Maestri, Alan Gallingani, Massimo De Filippo, Domenico Corradi

**Affiliations:** 1 Department of Cardiology, Parma University Hospital, Via Gramsci 14, 43125 Parma, Italy; ngaibazzi@gmail.com (N.G.); d.tuttolomondo@hotmail.it (D.T.); dott.danielesartorio@gmail.com (D.S.); 2 Department of Cardiac Surgery, Parma University Hospital, Via Gramsci 14, 43125 Parma, Italy; francesco.nicolini@unipr.it (F.N.); fmaestri@ao.pr.it (F.M.); agallingani@ao.pr.it (A.G.); 3 Unit of Pathology, Department of Medicine and Surgery, Parma University Hospital, Via Gramsci 14, 43125 Parma, Italy; alessandro.tafuni@unipr.it (A.T.); domenico.corradi@unipr.it (D.C.); 4 Department of Radiology, Parma University Hospital, Via Gramsci 14, 43125 Parma, Italy; 5 Department of Medicine and Surgery, Section of Radiology, University of Parma, Maggiore Hospital, Via Gramsci 14, 43125 Parma, Italy; massimo.defilippo@unipr.it

**Keywords:** thoracic aortic aneurysm, computed tomography, perivascular adipose tissue attenuation, vascular inflammation

## Abstract

On computed tomography (CT) imaging, a peri-vascular adipose tissue attenuation (pVAT) measure has been proposed as a non-invasive correlate of inflammation in the coronary artery vessels, and a single research group provided histopathological demonstration of this radiological/pathological correspondence. Our group has shown that patients with surgical-grade ascending aorta (AA) aneurysm display higher pVAT compared with patients with smaller aneurysms or normal AA. Based on histopathological studies on coronary arteries, we speculated that this correlation may be related to a non-otherwise specified aortic inflammatory process. However, since adipose tissue around the AA is often scant, and there are no histopathological studies confirming such hypothesized association between higher pVAT and inflammation around the AA, we cannot exclude that this pVAT change is secondary to different mechanisms, unrelated to the actual presence of peri-vascular inflammation. We performed a retrospective clinical/radiological/pathological study in 78 patients who underwent AA surgery with the aim to correlate pre-operatory pVAT on CT with histopathological findings from the surgical specimens. Histopathological review and immunohistochemistry were performed on the surgical aortic samples. The AA adventitial/periadventitial adipose tissue had higher pVAT by an increasing collagen fiber deposition, which progressively makes the fat hypotrophic and, in the late stages of this process, it replaces the normal soft tissue composition in this location. In the ascending aorta, pVAT on CT imaging is probably not a proxy for the presence of current vascular inflammation, although it may track changes involving the progressive substitution of perivascular adipose cells by higher-pVAT tissues, mainly fibrotic replacement.

## 1. Introduction

Although thoracic aortic aneurysm (TAA) is frequently an indolent disease, it represents a life-threatening condition. More than half of patients with TAA are asymptomatic and most cases are discovered at the time of radiography, or computed tomography angiography (CTA) scan. The estimated incidence of TAA, which has increased over the past few decades, is around 16.3 cases in men and 9.1 cases/100,000 patient years in women [1]. From a pathologic standpoint, TAAs comprise two different types of disease: proximal to the *ligamentum arteriosum*, its pathogenesis is mostly non-atherosclerotic, while, distally to the ligament, atherosclerosis is the dominant process predisposing to aortic wall dilation [2]. Computed tomography imaging is one of the foremost methods in imaging thoracic aorta diseases, and it allows a comprehensive evaluation of TAAs in terms of morphology, extent, thrombosis, relationship to adjacent structures, and signs of acute or imminent rupture [3,4]. On CTA imaging, a peri-vascular adipose tissue attenuation (pVAT) measure has been originally proposed as a non-invasive correlate of inflammation in the coronary artery vessels, although only a single research group provided histopathological demonstration of this radiological/pathological correspondence [5,6].

The measurement of pVAT seems to be capable of detecting vascular inflammation in non-coronary vessels by standard CTA imaging [7,8,9]. Our group has recently shown that patients with surgical-grade ascending aorta (AA) aneurysm display significantly higher pVAT compared with the ones with smaller aneurysms or even normal-diameter AA. Based on histopathological studies on coronary arteries, we speculated that this correlation may be related to a non-otherwise specified aortic inflammatory process [10].

However, since the presence of adipose tissue around the AA is often quantitatively scant, and there are no histopathological studies confirming the hypothesized association between higher pVAT measure and inflammation around the AA, it cannot be ruled out that this pVAT change is secondary to different mechanisms, unrelated to the actual presence of peri-vascular inflammation. On this basis, we performed a clinical/radiological/pathological study in patients undergoing AA surgery with the aim to correlate pre-operatory pVAT findings on CTA with the corresponding histopathological aspects obtained from the relevant surgical specimens. 

## 2. Materials and Methods

### 2.1. Patients

We retrospectively selected patients fulfilling all the following inclusion/exclusion criteria, in the time window between January 2011 and December 2018: 

Inclusion criteria: (I) surgical-grade AA aneurysm, where AA replacement was the primary indication to surgery, AA aneurysms where defined surgical-grade if CTA showed localized dilation of the AA with maximal diameter >54 mm (for patients with tricuspid aortic valve) or >45 mm (for patients with a bicuspid aortic valve); (II) planned surgery was carried out within 2 months after the index CTA scan; (III) availability of both the original histopathological slides and the relevant tissue blocks for pathological assessment of the excised ascending aorta sample.

Exclusion criteria: (I) detectable or known conditions capable of inducing local or systemic fibrosis or inflammation possibly unrelated to the specific AA disease, such as prior myocardial infarction, prior heart/aortic surgery (valvular or ascending aorta surgery, coronary revascularization or any other type of percutaneous intervention), current or recent acute/chronic inflammatory/infective diseases, and malignant tumors; (II) known Marfan’s syndrome; (III) CTA with artifacts affecting pVAT measurement.

### 2.2. Computed Tomography Angiography

All studies were performed on a second-generation 128-slice dual-source computed tomography system (Definition Flash, Siemens Healthcare, Forchheim, Germany). All scans were carried out in a cranio-caudal direction with a prospective electrocardiogram (ECG)-triggered Flash protocol. Contrast-enhanced scans were performed from the thoracic inlet to the pubic symphysis. Scanning parameters for both groups were as follows: slice collimation of 128 × 0.6 mm with a z-flying focal spot, gantry rotation time of 280 ms, pitch of 3.2, and tube voltage of 100 kV (low-iodine group) or 120 kV (high-iodine group). A test bolus of 15 mL of CM followed by 30 mL of saline was used to evaluate the scan delay at the middle of the descending aorta. Monitoring scans (with a temporal resolution of 1 s) began to be acquired 5 s after the start of the injection. The optimal scan delay time was calculated by adding the peak enhancement time from the monitoring scan to 10 s. The actual image was acquired by using 1 mL of contrast medium per kg body weight, followed by 30 mL of saline solution. The injection rate of contrast medium and saline solution was 4 mL/s for all subjects. Computed tomography angiography images were reconstructed by a conventional filtered back projection (FBP) algorithm with a medium smooth kernel designed for cardiac imaging (B26f). In the low-iodine group, images were also reconstructed by a sinogram-affirmed IR algorithm (SAFIRE, Siemens Healthcare, Erlangen, Germany) with the corresponding vascular kernel (I26f). With the IR algorithm as recommended by manufacturer, a medium strength of 3 was used.

### 2.3. Peri-Aortic Adipose Tissue Attenuation

**Figure 1:**
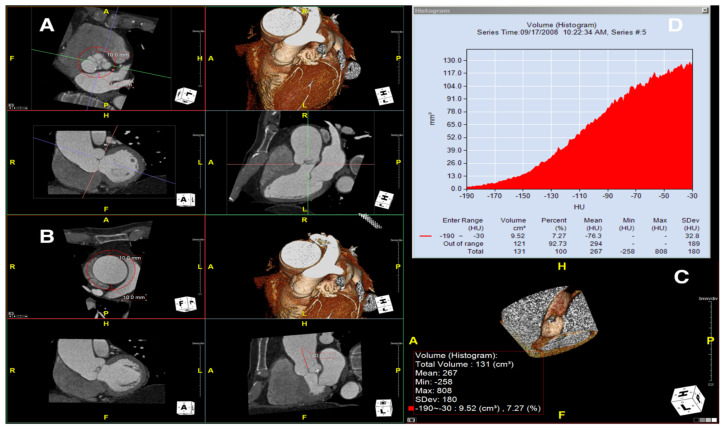
Step-by step process used to measure peri-aortic adipose tissue attenuation at Computed Tomography imaging. After spatial planes reorientation along the long and short axis of the ascending aorta, a 2D circle using an incremental +10 mm radius beyond the aortic diameter at the cusps level is traced (**A**) and a second circle is then drawn 40 mm distally, using in this case the diameter of the aorta at that level (**B**). The software automatically interpolates the volume between the two circles and builds the 3D volume sample (**C**), whose volume is known (131 cm^3^ in this case). The software also calculates the volume corresponding to a pre-determined Hounsfield Unit range; for fat sampling we used a −190 to −30 Hounsfield units (HU) range (in this case a volume of 9.52 cm^3^ is automatically measured). Panel (**D**) (fat attenuation histogram) shows that the mean attenuation of fat found in this custom sample volume shown in panel C is −76.3 HU.

To measure the periaortic pVAT in the ascending aorta, we used a software package (Aquarius Workstation® V.4.4.13, TeraRecon Inc., Foster City, CA, USA) that allowed us to trace volume samples (see Figure 1 showing how the step-by-step post-processing method was applied to measure mean pVAT). In this case, after reorientation of the planes to obtain long and short axis of the ascending aorta, the aortic short axis plane was used to draw a volume sample interpolated between two traced circles, the first at the level of the aortic valve/cusps and the second exactly 40 mm upper (distally) along the aortic course; both circles were traced with a radius equal to the aortic radius (at that specific level) +10 mm (measured as a radial distance of 10 mm from the outer aortic wall border). Peri-vascular adipose tissue in the volume sample was automatically calculated by the software based on the attenuation histogram of peri-vascular adipose tissue within the range of −190 Hounsfield units (HU) to −30 HU, as described previously [10]. We ascertained the perivascular pVAT by quantifying the weighted perivascular fat attenuation after adjustment for technical parameters (if 100 kV voltage was used instead of standard 120 kV voltage, the mean HU value was corrected dividing by 1.11485). Adipose tissue contained in the drawn volume sample, an irregular hollow cylinder, was automatically measured for both fat volume and mean pVAT ± standard deviation (SD). 

### 2.4. Histopathology

**Figure 2:**
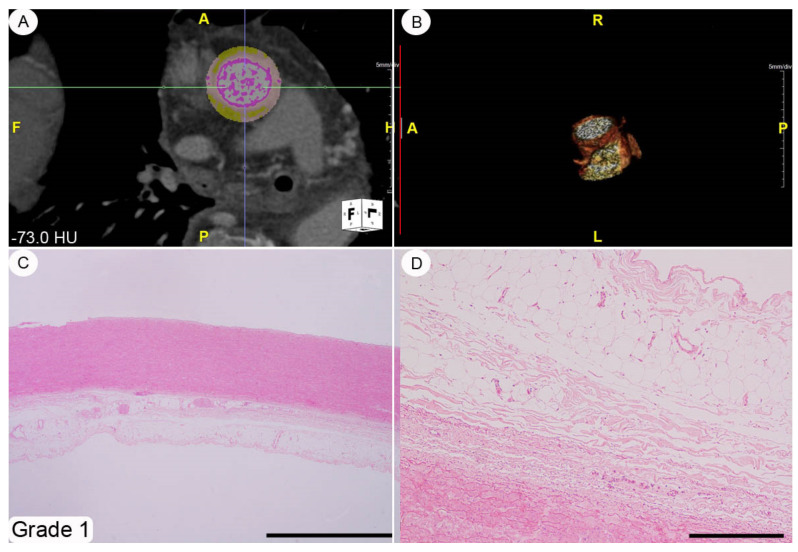
Adventitial fibrosis, grade 1. This index case refers to a 72 year-old male patient suffering from AA aneurysm characterized by an attenuation histogram of peri-vascular adipose tissue of mean −73 HU (CTA shown in (**A**,**B**)). The histopathological correlates of CTA findings show an aortic *tunica media* thickness within the normal ranges (**C**) and an initial collagen fiber deposition affecting the adipose tissue of the adventitial and peri-adventitial layers (**D**) with focal signs of consequent adipocyte hypotrophy/atrophy (**D**). Staining. (**C**,**D**): hematoxylin-eosin. Original magnifications. (**C**): ×2 (bar is 1 mm), (**D**): ×10 (bar is 250 um).

**Figure 3:**
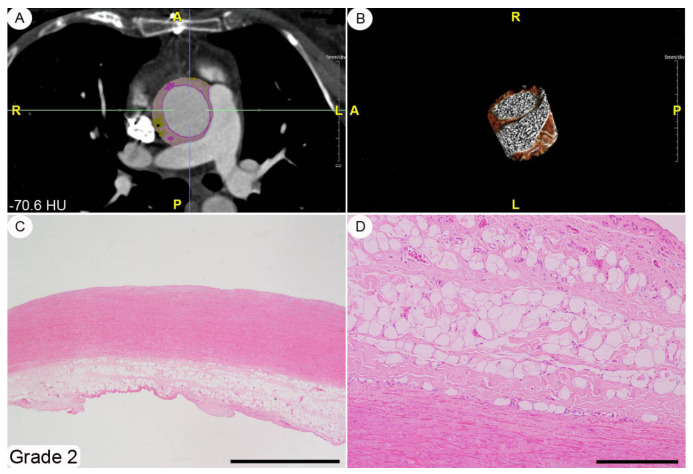
Adventitial fibrosis, grade 2. This index case refers to a 69-year-old male patient with AA aneurysm on CTA, an attenuation histogram of peri-vascular adipose tissue of mean −70.6 HU (**A**,**B**). The corresponding histopathology displays a mild-to-moderate increase in *tunica media* thickness (**C**) and a moderate replacement of the adventitial/periadventitial soft tissues by collagen fibers (**D**) with more obvious aspects of adipocyte hypotrophy/atrophy as in Grade 1 (**D**). Staining. (**C**,**D**): hematoxylin-eosin. Original magnifications. (**C**): ×2 (bar is 1 mm), (**D**): ×10 (bar is 250 nm).

**Figure 4:**
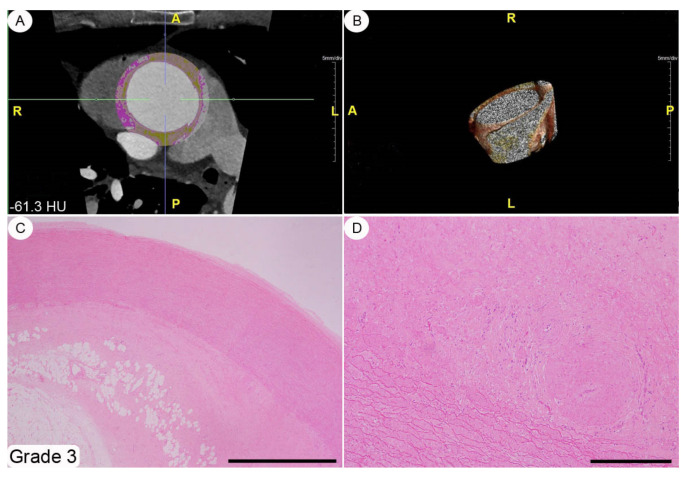
Adventitial fibrosis, grade 3. This index case refers to a 71-year-old male/female patient with AA aneurysm with, an attenuation histogram of peri-vascular adipose tissue of mean −61.3 HU (**A**,**B**). The resultant histopathology shows a moderate increase in *tunica media* thickness (**C**) as well as a severe fibrosis of the adventitial adipose tissue with a trespassing on the periadventitial soft tissues. Only sparse adipose tissue islands are detectable in the adventitial/periadventitial spaces (**C**). The fibrotic tissue is dense and scarcely cellular (**D**). Staining. (**C**,**D**): hematoxylin-eosin. Original magnifications. (**C**): ×2 (bar is 1 mm), (**D**): ×10 (bar is 250 nm).

A histopathological review of the original surgical aortic samples was performed for all the patients enrolled in the present study. This re-analysis was aimed at confirming the original histological evaluation and, in particular, to provide a semiquantitative assessment of the various microscopic changes that had occurred in the aortic wall. At the time of surgery, the aortic samples (average size about 4 × 3 cm^2^ and spatially oriented by silk sutures of different colors) were fixed in a 10% buffered formalin solution for 24–48 h and, subsequently, embedded in paraffin tissue blocks, from which 3–4 mm-thick sections were obtained. These sections were stained with hematoxylin-eosin by following routine methods. The histopathological modifications affecting the thoracic aortic wall we chose to explore (i.e., adventitial fibrosis, architectural disarray of the medial elastic component, and atherosclerotic degeneration) were semi-quantitatively assessed using 0-to-3 grade scales, as absent, mild, moderate, and severe change, respectively (see Figure 2, Figure 3, Figure 4 and caption for a more detailed description of the various grades). These morphological evaluations were carried out by two pathologists (DC and AT) blinded to patient characteristics. The adventitial inflammatory infiltrate was evaluated semi-quantitatively by means of a 0-to-3 grade scale as absent, focal, multifocal or diffuse.

### 2.5. Immunohistochemistry

An immunohistochemical analysis was performed to assess the *vasa vasorum* density and the extent of an inflammatory infiltrate. The blood vessels were identified by testing an anti-CD31 primary antibody (LifeSpan BioSciences, Seattle, WA, USA; code LS-B3446, monoclonal; dilution 1:400). In each case, the number of *vasa vasorum* profiles (with the exclusion of capillaries) was counted along the entire adventitial profile (always >1 cm) at a magnification of ×20 (microscopic field diameter: 1.10 mm). The resulting vascular density per mm of aortic adventitia was obtained by dividing their absolute number by the observed adventitial length (in mm). The inflammatory infiltrate was typified by testing the following primary antibodies: anti-CD3 (Invitrogen, South San Francisco, CA, USA; code MA5-12577, monoclonal; dilution 1:20), anti-CD4 ((LifeSpan BioSciences, Seattle, WA, USA; code LS-B9816, monoclonal, dilution 1:100), anti-CD8 (Invitrogen, South San Francisco, CA, USA; code MA1-80231, monoclonal, dilution 1:50), anti-CD20 (LifeSpan BioSciences, Seattle, WA, USA; code LS-B8609, monoclonal; dilution 1:50), anti-CD138 (Invitrogen, South San Francisco, CA, USA; code MA5-12400, monoclonal; dilution 1:20), anti-CD163 (Invitrogen, South San Francisco, CA, USA; code MA5-11458, monoclonal; dilution 1:40), anti-myeloperoxidase (LifeSpan BioSciences, Seattle, WA, USA; code LS-B3899, monoclonal; dilution 1:200). The extent of the global inflammatory infiltrate was semi-quantitatively assessed using a 0-to-3 grade scale, as absent, mild, moderate, and severe.

### 2.6. Statistical Analysis

Data with normal distribution were expressed as mean and standard deviation, while data not normally distributed were expressed as median and interquartile range. To compare normally distributed data, t-test for independent samples was used, and Mann–Whitney-U test was used for data not normally distributed. Categorical data were expressed as number and percentages and compared with Fisher exact test. The association between two continuous parameters was assessed with simple linear correlation analysis and Kendall’s tau was used to test the association between continuous and discrete ordinal parameters. Analysis of variance (ANOVA) was used to compare pVAT values among subgroups divided based on fibrosis or other histopathologic findings. Levene’s test was used to assess homogeneity of variance; if homogeneity was not found, Welch’s ANOVA was preferred for analysis. Tukey HSD test was used in the case of homogeneity of variance, otherwise Games–Howell test was used. Two-sided p is always reported. All analyses were made using SPSS software, version 23.0 or Statsdirect software, version 3.0 (http://www.statsdirect.com, accessed on 20 July 2021. StatsDirect Ltd., Birkenhead, UK, 2013).

## 3. Results

### 3.1. Patient Population

Eighty-one patients fulfilled the inclusion criteria and were enrolled in this study; three patients fulfilling the inclusion criteria were subsequently excluded on the basis of one exclusion criterion, namely, known inflammatory bowel disease (*n* = 1), rheumatoid arthritis (*n* = 1), and known Marfan’s syndrome (*n* = 1). The overall demographics, clinical details, risk factors, and CTA data of the final 78 patients who were included in this study are summarized in Table 1. Mean age was 61 ± 14 years, 20 were females (26%), and 34 patients (44%) had an associated bicuspid aortic valve. Mean pVAT was −68.5 ± 6.4 HU.

### 3.2. Histopathology

**Figure 5:**
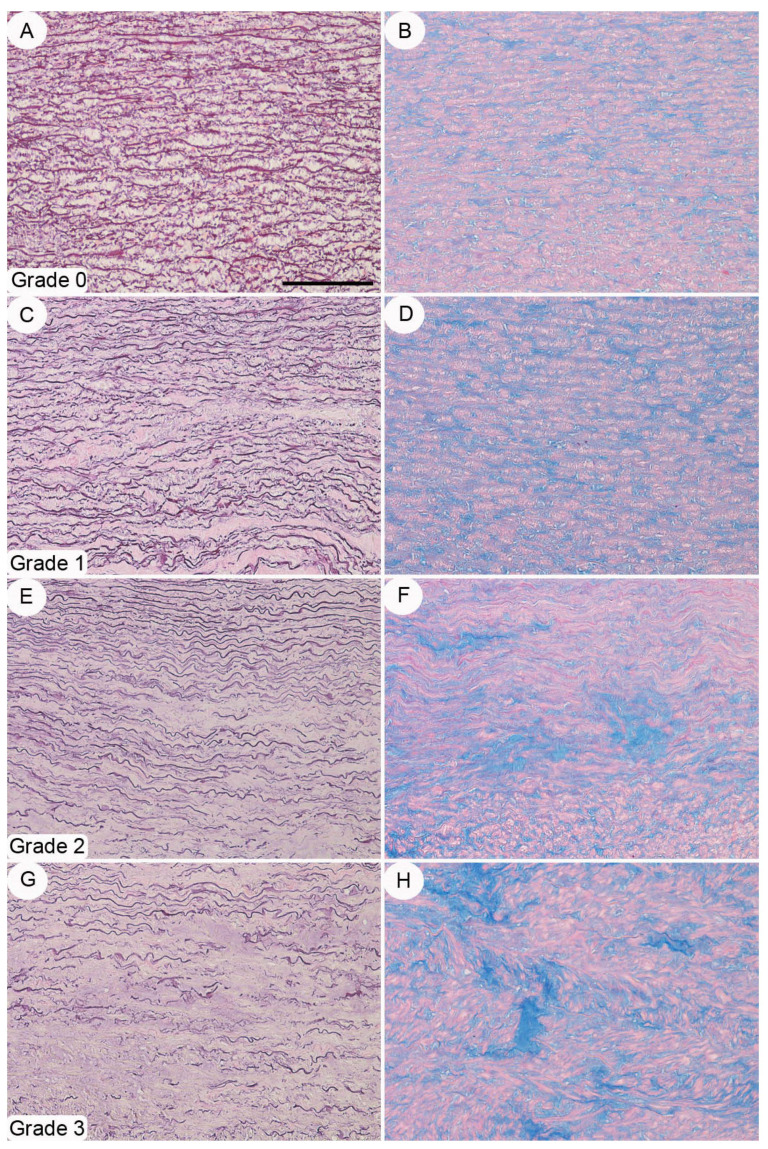
Aortic elastic fiber component disruption grading. (**A**,**B**), Grade 0 characterized by a normal distribution of the elastic fibers belonging to the aortic *tunica media* and no alcianophilic bluish accumulations. (**C**,**D**). Grade 1 with fragmentation of single elastic fibers and alcianophilic material still within the normal limits. (**E**,**F**). Grade 2 with fragmentation of adjacent elastic fibers and mild accumulation of alcianophilic material. (**G**,**H**). Grade 3 characterized by diffuse disruption of elastic fibers and moderate-to-severe accumulations of alcianophilic material. Staining. (**A**,**C**,**E**,**G**): Weigert’s elastic stain; (**B**,**D**,**F**,**H**): alcian blue stain. Original magnification. A–H: ×20 (bar is 150 um).

A histopathological review of the original aortic slides that had been stained with hematoxylin-eosin showed changes of varying amounts affecting the adventitia; qualitatively, these modifications ranged from a very mild collagen deposition to a general replacement of the adventitial soft tissues by a scar-like and scarcely cellular fibrous extracellular matrix (see Figure 2, Figure 3, Figure 4 for grading examples). No cases of inflammatory aortic aneurism were diagnosed within the patient population enrolled in this study [11]. The aortic *tunica media* was always modified by structural changes affecting both the elastic and smooth-muscle components. In particular, the elastic fibres showed different degrees of fragmentation, and, in the most severe cases fibrosis and/or lacunae filled by alcianophilic material in keeping with glycosaminoglycans (Figure 5). Accordingly, in parallel with elastic fibre fragmentation and fibrosis, the smooth-muscle cell component became progressively less dense. However, no aortic samples with primitive large smooth-muscle cell necrosis were detected [12]. Varying degrees of histopathological modifications in the setting of atherosclerosis had taken place. These ranged from subtle subendothelial thickenings, to non-complicated plaques, and, finally, to complicated plaques also involving different *tunica media* layers (Supplementary Figure S1). The semi-quantitative assessment of adventitial fibrosis, architectural disarray of the medial elastic component, and atherosclerotic degeneration are reported in Supplementary Table S1. 

### 3.3. Immunohistochemistry

In the AA, the adventitial blood vessel density ranged from 7.08 to 12.09 per mm^2^ of adventitial tissue (Supplementary Figure S2). In particular, with regard to adventitial fibrosis, in Grade 0 this density averaged 9.7 ± 1.2, in Grade 1 8.6 ± 1.6, in Grade 2 8.3 ± 2.3, and in Grade 3 9.2 ± 0.9, with no statistically significant differences between the three pathological groups as well as between these and the controls. The semi-quantitative assessment of the inflammatory infiltrate is reported in Supplementary Table S1. When detectable, the adventitial inflammatory component was of chronic type and mainly composed of B and T lymphocytes, with this latter subset being principally constituted of CD4^+^ cells. Only scattered plasma cells and histiocytes completed the histopathological picture (Supplementary Figure S3). 

### 3.4. Correlation between Peri-Aortic Fat Attenuation and Clinical/CTA/Histopathological Findings

**Figure 6:**
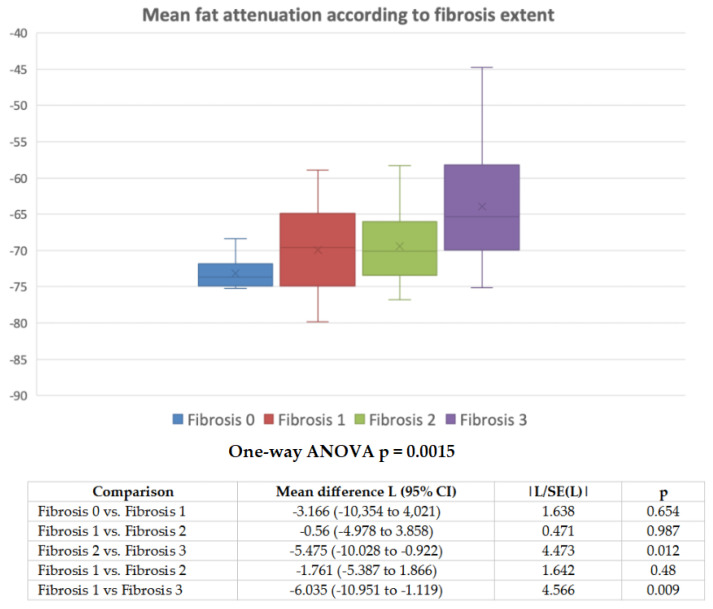
Mean fat attenuation in each group of patients divided based on fibrosis grade and *p* for significance of one-way analysis of variance (Welch adjusted ANOVA), with Tukey–Kramer multiple comparisons.

**Figure 7:**
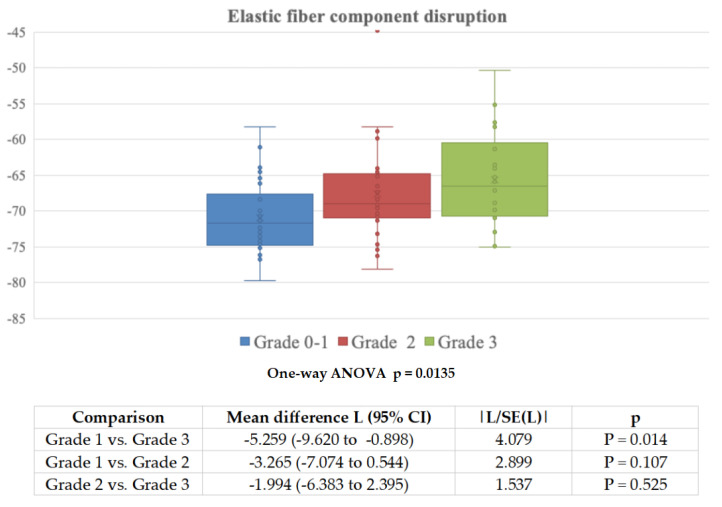
Mean fat attenuation in each group of patients divided based on elastic fibers disruption grade and *p* for significance of one-way analysis of variance (Welch adjusted ANOVA), with Tukey–Kramer multiple comparisons.

On univariate statistical analysis, only the maximum aneurysm diameter (r = −0.372, 95% CI 0.161 to 0.55, *p* < 0.001) and peri-aortic fat volume (inversely correlated, r = −0.432, 95% CI −0.598 to −0.231, *p* < 0.001) among variables measurable on CTA, were significantly correlated with pVAT. Among the histopathologic variables the adventitial/periadventitial fibrosis grading (ANOVA *p* = 0.0015; see Figure 6) and medial elastic fiber disruption grading (ANOVA *p* = 0.0135; see Figure 7) were significantly correlated with pVAT. Available clinical variables or other histopathologic findings (atherosclerotic plaque grading or the adventitial vascular profile density) did not significantly correlate with pVAT. Further, no statistical correlation was found between the degree of inflammatory infiltrate and pVAT. However, on multiple linear regression analysis, only the grade of fibrosis (r = 0.304, *p* = 0.011) and the peri-aortic fat volume on CTA (inversely correlated, r = −0.574, *p* < 0.001) proved to be independent predictors of pVAT. Supplementary Table S2 reports the univariable association of clinical/CTA/histopathological parameters with the grade of adventitial/periadventitial fibrosis, showing that only pVAT, maximum aneurysm diameter, and cigarette smoking were significantly associated with higher grade of fibrosis at histopathology.

Intraclass correlation coefficient between two separate sets of aortic pVAT measurements performed by the same operator on 30 random exams has been recently reported by our group as an intra-class correlation coefficient =0.943, while the same parameter assessed between two different readers, for the same 30 exams, was 0.904; such values of intra- and inter-rater agreement are generally defined as excellent.

## 4. Discussion

In this manuscript we have investigated, in the ascending aorta district, the structural correlate of what has previously been defined by Antonopulos et al. as “CTA fat attenuation index” in the coronary artery perivascular adipose tissue [5]. These authors stated that the CTA fat attenuation signal was the result of decreased adipocyte lipid content and size, both of these being secondary to an inflammatory process in the setting of atherosclerosis in the coronary arteries. On this basis, our group has recently observed that similar pVAT findings could be detected in the adventitial/periadventitial AA soft tissues from patients suffering from, and surgically treated for, TAAs [10]. However, since the routine pathological practice on the signing out of such anatomical specimens was mostly against a flogistic process able to significantly modify the adventitial milieu, we decided to explore which histopathological parameters the pVAT was correlated with, in this aortic aneurysm setting. The most relevant results of this study are: (1) the ascending aorta adventitial/periadventitial adipose tissue was “attenuated” by an increasing collagen fiber deposition, which progressively makes the fat hypotrophic, atrophic, and, finally, in the late stages of this process, it replaces the normal soft tissue composition in this location (see the inverse correlation of pVAT with fat volume on CTA); (2) on a multivariate statistical analysis, pVAT did not correlate with either a disarrangement of the elastic fiber component of the aortic wall or the severity of the atherosclerotic plaques; (3) pVAT did not associate with a loss of the adventitial vascular profiles; (4) at the time of surgery, the adventitial inflammatory infiltrate was in the overwhelming majority of cases very mild and, overall, it did not statistically correlate with pVAT.

Based on everyday pathological experience, and confirmed by the results of this investigation, the measurement of pVAT of the TAAs adventitial/periadventitial fat on CTA refers to a progressive replacement of the adipocyte lobules by a different tissue/extracellular matrix with a consequent shift of its specific mean X-ray attenuation (measured in Hounsfield Units) to a higher mean value. It is well known that the adipose tissue is particularly prone to prompt volume changes in terms of either hypotrophy or atrophy as a result of permeations by newly deposed harder tissues and/or infiltrations by different cell populations (e.g., inflammations, tumors) [13]. 

The biological/histopathological sequence of events that occur in the pathogenesis of TAA, is dominated by increasing grades of elastic lamellae fragmentation with pooling of proteoglycans, with appearance of cyst-like structures and loss of nuclei in the media (so-called “medial degeneration” and “medionecrosis”, respectively). In our series of cases (all of them belonging to a late-stage (surgical) phase of the disease) no significant signs of inflammation were found in the adventitial/periadventitial soft tissues. The only increasing tissue modification was a non-specific deposition of collagen fibers, which started from the adventitia and, with the disease progression, extended into the periadventitial soft tissues; this fact caused a progressive decrease in fat and, finally, resulted in higher attenuation/density of the adipose tissue signal on CTA scan. In most cases the inflammatory infiltrate was very mild and did not correlate with either pVAT or the fibrotic adventitial change. Unlike what was described in terms of coronary artery compartment atherosclerosis, where pVAT was apparently associated with the degree of inflammation, the AA aneurysm pathophysiology was basically not atherosclerotic in nature [5,14,15,16].

We cannot definitively rule out that, in the long pre-surgical phase of this disease, chronic inflammation could have been a more significant process in the outer aortic circumference; the histopathological analysis could have been simply conducted at a later stage, when only the end product of long-standing inflammation (fibrosis) could be demonstrated. However, even in the aortic specimens characterized by the mildest adventitial changes, which are supposed to represent an earlier stage of the pathogenetic spectrum, a significant inflammatory infiltrate was not detectable. The degree of elastic fiber disruption was also statistically correlated with the adventitial fibrosis; however, their fragmentation did not represent an independent predictor of mean pVAT on multivariate statistical analysis. 

Contrary to what was originally supposed, the adventitial vascular profile density did not decrease in parallel with the increasing perivascular fibrosis. This result makes a preponderant ischemic process less likely in the establishment, or maintenance, of the aortic wall changes in the setting of TAA.

To conclude, with this clinical/radiological/pathological investigation we have demonstrated that, at the latest (surgical) stages of TAA, the aortic adventitial soft tissues are modified by a progressive fibrotic replacement which, in this setting, is the relevant pathological correlate of so-called “peri-vascular fat attenuation” on pre-surgical CTA scan. In all likelihood, in TAAs, this higher adipose tissue radiodensity, which is identified on the basis of its typical HU range, is, on the one hand, the result of both a gradual disappearance of the fat tissue lobules and, on the other hand, secondary to the replacing fibrotic component that resets the CTA signal at an overall higher HU level.

## 5. Conclusions

In the ascending aorta, perivascular fat tissue attenuation on CTA imaging is probably not a proxy for the presence of current vascular inflammation, although it may track changes involving a progressive substitution of perivascular adipose cells by higher attenuation tissues, mainly fibrotic replacement.

## Figures and Tables

**Table 1:** Demographic, clinical, and main CTA and histopathology characteristics of the study group.

**Demographics *n* = 78**	
Age, mean (SD), years	61 (14)
Female gender, *n* (%)	20 (26)
BMI, median (lower-upper quartile), kg/m^2^	28 (5)
**Clinical Risk Factors**	
Hypertension, *n* (%)	51 (66)
Hypercholesterolemia, *n* (%)	18 (23)
Current Smoker, *n* (%)	27 (34)
Diabetes mellitus, *n* (%)	10 (13)
**Drug Therapy**	
Statin, *n* (%)	22 (28)
Beta-blocker, *n* (%)	32 (41)
**Computed Tomography Angiography**	
Bicuspid valve, *n* (%)	34 (44)
Ascending aorta diam, mean (SD), cm	5.3 (0.7)
Fat volume, mean (SD), cm^3^	8.5 (5.3)
pVAT, mean (SD), Hounsfield Units	−68.5 (6.4)
**Histopathology**	
Fibrosis grade, median (interquartile range), 0–3 semiquantitative scale	2 (1.75)
Elastic component disruption, median (interquartile range), 0–3 semiquantitative scale	2 (1.75)

BMI, body mass index; pVAT, peri-vascular adipose tissue attenuation.

## Supplementary Materials

The following are available online at https://www.mdpi.com/article/10.3390/diagnostics11101799/s1, Figure S1: Aortic atherosclerotic plaque grading, Figure S2: Adventitial inflammatory infiltrate, Figure S3: Aortic adventitia blood vessels (*vasa vasorum*), Table S1: Semi-quantitative assessment of the histopathological changes, Table S2: Association of main demographic, clinical, and computed tomography attenuation characteristics versus the fibrosis grade at histopathology in the study group.
