# Supplementary material for: The Histopathological Correlate of Peri-Vascular Adipose Tissue Attenuation on Computed Tomography in Surgical Ascending Aorta Aneurysms: Is This a Measure of Tissue Inflammation?
Source: Diagnostics (Basel). 2021 Sep 29;11(10):1799. doi: 10.3390/diagnostics11101799 (PMC8535015; doi:10.3390/diagnostics11101799)
Supplement: Supplementary file 1 [file diagnostics-11-01799-s001.zip › diagnostics-1335122-supplementary.pdf]

The Histopathological Correlate of Peri-Vascular Adipose Tissue Attenuation on Computed Tomography in Surgical Ascending Aorta Aneurysms: Is This a Measure of Tissue Inflammation?

Nicola Gaibazzi [1,†], Domenico Tuttolomondo [1,†], Francesco Nicolini [2], Alessandro Tafuni [3], Daniele Sartorio [1], Chiara Martini [4,*], Francesco Maestri [2], Alan Gallingani [2], Massimo de Filippo [5] and Domenico Corradi [3]

[1] Department of Cardiology, Parma University Hospital, Via Gramsci 14, 43125 Parma, Italy; ngaibazzi@gmail.com (N.G.); d.tuttolomondo@hotmail.it (D.T.); dott.danielesartorio@gmail.com (D.S.)

[2] Department of Cardiac Surgery, Parma University Hospital, Via Gramsci 14, 43125 Parma, Italy; francesco.nicolini@unipr.it (F.N.); fmaestri@ao.pr.it (F.M.); agallingani@ao.pr.it (A.G.)

[3] Department of Medicine and Surgery, Unit of Pathology, Parma University Hospital, Via Gramsci 14, 43125 Parma, Italy; alessandro.tafuni@unipr.it (A.T.); domenico.corradi@unipr.it (D.C.)

[4] Department of Radiology, Parma University Hospital, Via Gramsci 14, 43125 Parma, Italy;

[5] Department of Medicine and Surgery, Section of Radiology, University of Parma, Maggiore Hospital, Via Gramsci 14, 43125 Parma, Italy; massimo.defilippo@unipr.it

† These authors contributed equally to this work as first author

* Correspondence: MartiniC@ao.pr.it; Tel.: +39-3457245174

Supplementary Materials

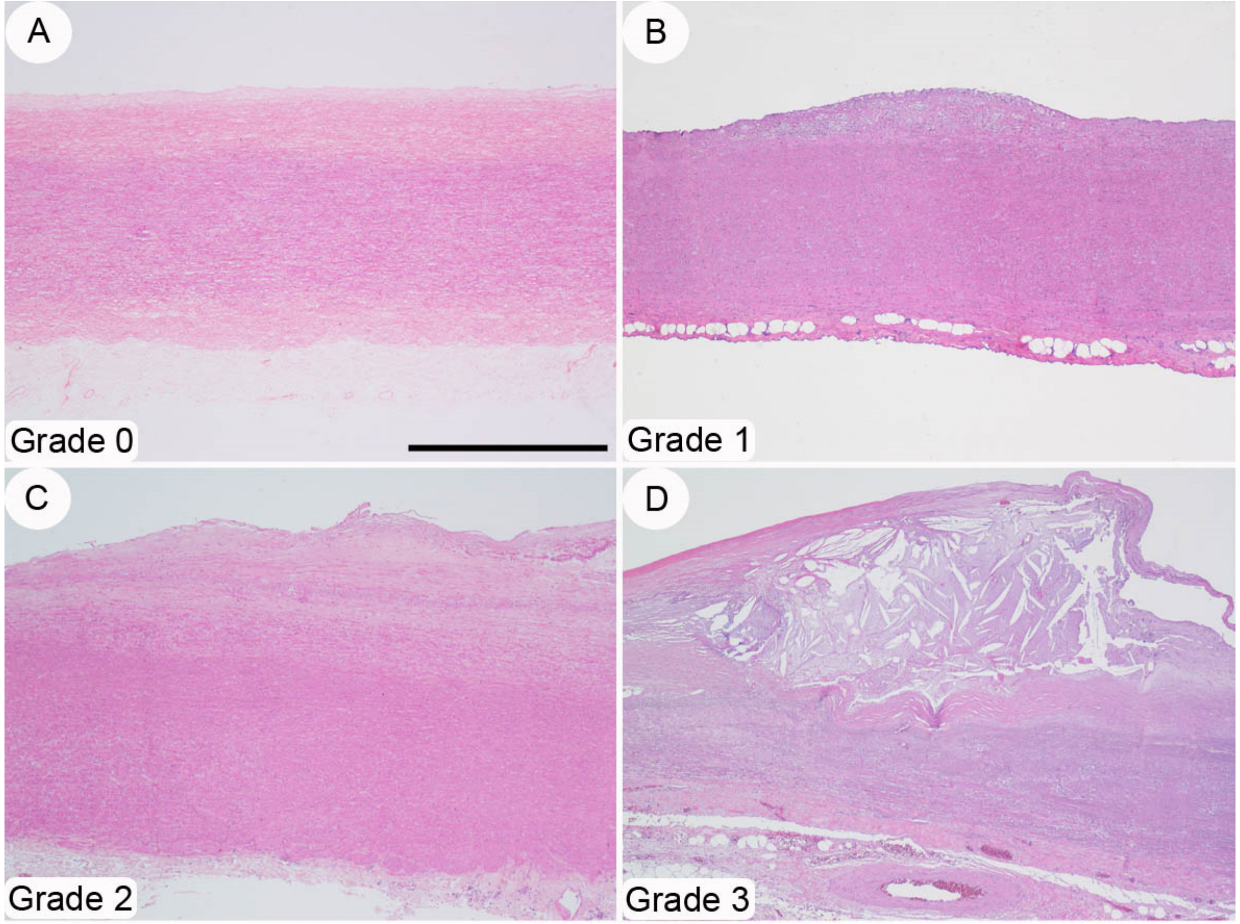

Figure S1. Aortic atherosclerotic plaque grading. **A**. Grade 0, control autopsy aorta obtained from the same anatomical location as that from surgical specimens. The intimal thickness is within the normal limits for age-matched individuals. **B**. Grade 1, there are some isolated plaque-like thickenings of the intimal layer in terms of fibrous tissue deposition and very mild inflammatory infiltrate (arrow). **C**. Grade 2, the plaques are larger and more diffuse than those in Grade 1 with sparse areas where lipidic material had been accumulated (arrow). **D**. Grade 3, the plaques are even larger than those in Grade 2 and contain significant amounts of lipidic material (arrow), inflammatory foci, and, often, spill into the *tonaca media* (arrowhead). Often, these plaques were ulcerated. Staining. A–D: hematoxy-lin-eosin. Original magnifications. A–D: ×4 (bar is 1 mm).

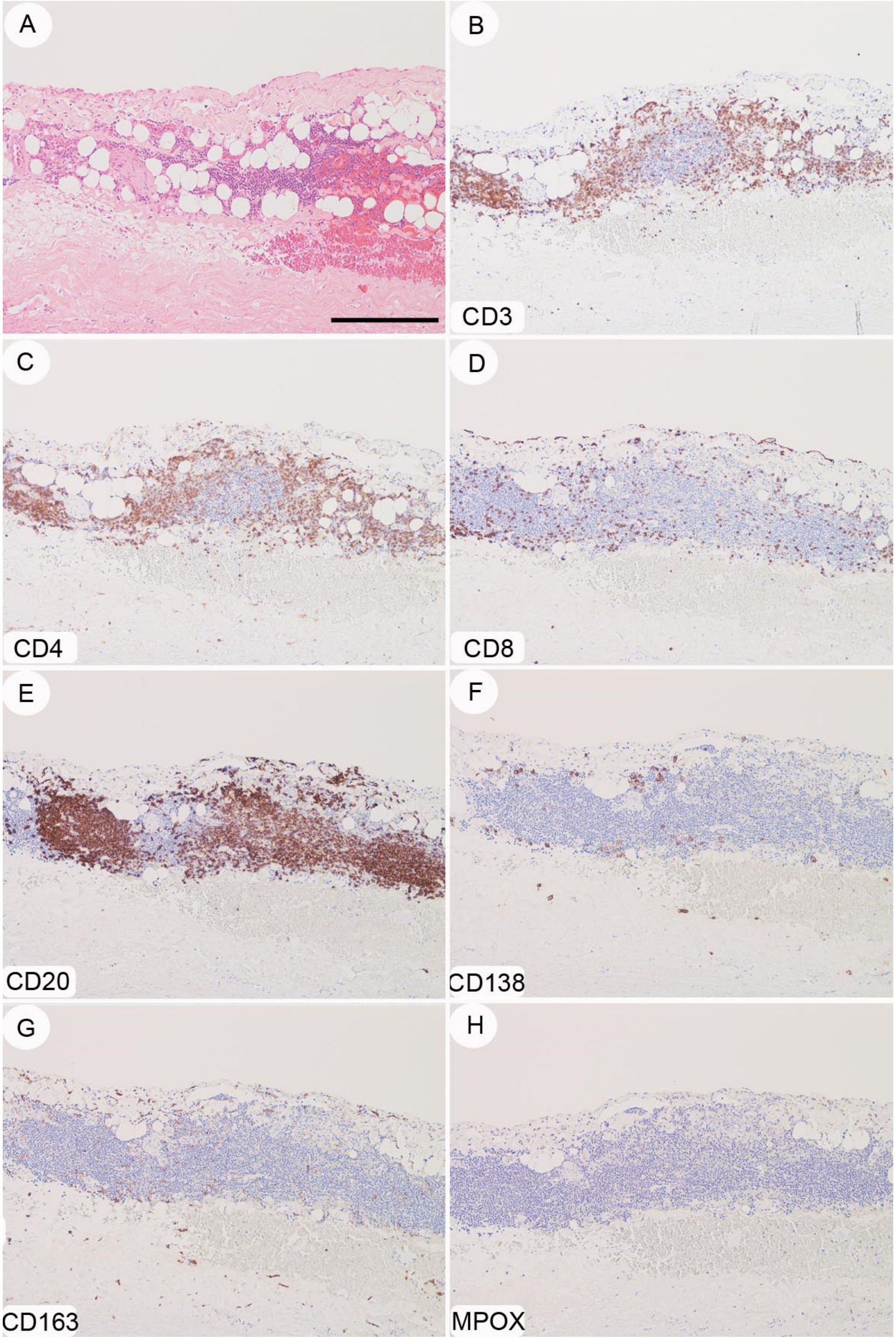

Figure S2. Adventitial inflammatory infiltrate. (**A**). Medium-power histopathological view of an aortic adventitia with a Grade 2 inflammatory infiltrate. Its immunohistochemical typing has been performed by investigating the following markers: CD3 (**B**), CD4 (**C**), CD8 (**D**), CD20 (**E**), CD138 (**F**), CD163 (**G**), myeloperoxidase (**H**). Staining. A: hematoxylin-eosin; B–H: immunohistochemical reaction revealed by 3,3′-Diaminobenzidine (DAB) and mildly counterstained by Harris hematoxylin. Original magnifications. A–H: ×10 (bar is 250 um).

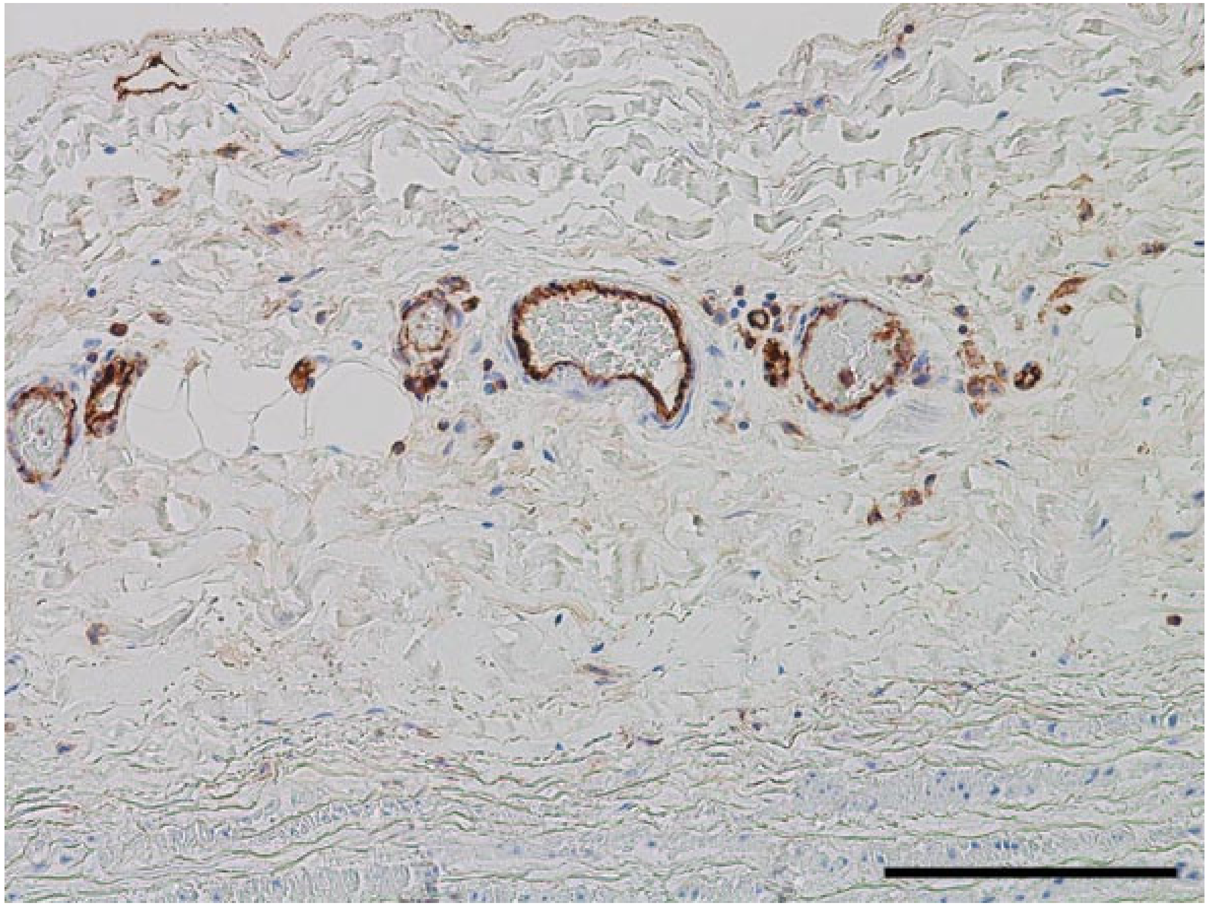

Figure S3. Aortic adventitia blood vessels (*vasa vasorum*). In this microphotograph, the aortic adventitia blood vessels (*vasa vasorum*) are immunohistochemically decorated after testing an anti-CD31 primary antibody (brown color). The asterisk indicates the *tonaca media* while the arrow the adventitial soft tissues. Staining: immunohistochemical reaction revealed by 3,3′-Diaminobenzidine (DAB) and mildly counterstained by Harris hematoxylin. Original magnification: ×20 (bar is 150 um).

Table S1. Semi-quantitative assessment of the histopathological changes.

	Grade 0	Grade 1	Grade 2	Grade 3
Adventitial fibrosis, n. of cases (%)	6 (8)	22 (28)	31 (40)	19 (24)
Medial disarray, n. of cases (%)	2 (3)	27 (35)	30 (38)	19 (24)
Atherosclerotic plaques, n. of cases (%)	57 (73)	9 (12)	5 (6)	7 (9)
Adventitial inflammatory infiltrate, n. of cases (%)	9 (12)	66 (85)	3 (4)	0

Table S2. Association of main demographic, clinical, and computed tomography attenuation characteristics versus the fibrosis grade at histopathology in the study group. Kendall’s rank correlation is additionally reported for continuous variables unlikely to be from a normal distribution.

	Simple Linear Regression Correlation, r (95% CI)	2 Sided *p*	Mann-Whitney U Test, Theta (95% CI)	2 Sided *p*	Kendall's Rank Correlation, Tau (95% CI)	2 sided *p*
**Demographics**						
Age	0.148 (−0.08 to 0.361)	0.203				
Sex			0.517 (0.372 to 0.658)	0.839		
Body mass index	0.087 (−0.143 to 0.308)	0.459				
**Clinical Risk Factors**						
Hypertension			0.349 (0.196 to 0.551	0.137		
Hypercholesterolemia			0.459 (0.307 to 0.622)	0.654		
Current Smoker			**0.324 (0.204 to 0.478)**	**0.020**		
Diabetes mellitus			0.47 (0.289 to 0.662)	0.834		
**Drug Therapy**						
Statin therapy			0.446 (0.298 to 0.607)	0.52		
**CTA**						
Bicuspid valve			0.528 (0.384 to 0.667)	0.691		
Ascending aorta diameter	**0.345 (0.130 to 0.530)**	**0.002**			**0.233 (0.093 to 0.372)**	**0.01**
Fat volume	0.143 (−0.085 to 0.356)	0.219			0.074 (−0.067 to 0.215)	0.402
pVAT	**0.397 (0.188 to 0.571)**	**<0.001**			**0.274 (0.139 to 0.409**	**0.001**

CTA, computed tomography angiography; pVAT, peri-vascular adipose tissue attenuation.
